# Potential Co-Factors of an Intraoral Contact Allergy—A Cross-Sectional Study

**DOI:** 10.3390/dj8030083

**Published:** 2020-08-03

**Authors:** Constanze Olms, Jana Schor, Maryam Yahiaoui-Doktor

**Affiliations:** 1 Private Practice, 29410 Salzwedel, Germany; 2 Max Planck Institute for Evolutionary Anthropology, Deutscher Platz 6, 04103 Leipzig, Germany; 3 Department of Molecular Systems Biology, Helmholtz Centre for Environmental Research GmbH—UFZ, 04103 Leipzig, Germany; jana.schor@ufz.de; 4 Institute for Medical Informatics, Statistics and Epidemiology (IMISE), University of Leipzig, 04103 Leipzig, Germany; maryam.yahiaoui@imise.uni-leipzig.de

**Keywords:** contact allergy, metal sensitization, hypothyroidism

## Abstract

The aim of this cross-sectional study was to evaluate the frequency of dental allergens and potential co-factors, especially hypothyroidism, for patients with an intraoral contact allergy. From 2015 to 2016, patients with confirmed symptoms of an intraoral contact allergy (study group SG n = 50) were recruited in the dental clinic of the University of Leipzig. The participants of the control group (CG n = 103) were patients without oral diseases or intraoral symptoms of a contact allergy. For the data collection, a new “Allergy questionnaire” was developed. Information on allergies and general diseases were collected. The statistical analysis was carried out with SPSS 23.0. Sensitizations/allergies to metals and composites were higher in SG compared to CG. Of all study participants (n = 148), 14.2% (n = 21) had a nickel allergy. In 18% (n = 8) of the SG a cobalt allergy based on all metal allergens could be seen. In addition, an association between a nickel and cobalt allergy was found. Hypothyroidism occurred significantly more frequently (*p* = 0.049) in SG than in CG. Sensitizations and allergies can occur to metals in dental alloys. Hypothyroidism increased the risk of having an allergy threefold.

## 1. Introduction

Dental materials are among the most common artificial materials that are incorporated into the human body. Intolerances to dental materials can either be directly associated with toxic damage or occur as part of sensitization or allergy. In recent decades, an increase in allergies could be observed in the world population [1,2,3,4,5,6,7]. This increase is attributed to lifestyle and cultural, economic, ecological and other factors [1]. In the German population, the number of allergies has been growing continuously for years [8]. Almost a third of the population in Germany is affected by an allergic disease in the course of their life and allergic sensitization has been found in 50% of the German population [8]. In the 2013 study on Adult Health in Germany (DEGS1), nearly 20% of the respondents said that they had an allergic disease in the last 12 months. The most common allergic diseases were hay fever (14.8%), bronchial asthma (8.6%) and contact dermatitis (8.1%) [9]. Contact eczema is also increasingly observed in children [10,11,12], and is also increasingly prevalent among the elderly [13,14]. The most important risk factors for a contact allergy are: workload, age, gender, the handling of consumer goods and a genetic predisposition [15]. Allergic contact dermatitis has thus reached a frequency similar to that of diabetes mellitus [16].

The introduction of new materials into dentistry also increases the likelihood of an allergic intolerance reaction as a result of dental treatment [17]. Current epidemiological studies on dentistry are lacking. A previous clinical case report shows that multiple allergies can be associated with a lichenoid oral mucosa lesion. In this case, a L-Thyroxin substituted hypothyroidism was identified [18]. An increase in hypothyroidism has also been described in the clinical picture of oral lichen planus and oral lichenoid lesions [19,20]. An analysis of data from 2012 to 2016 showed that one of the most common diseases in patients with intraoral symptoms of contact allergy is a thyroid disorder (32,6% n = 28/86) [21]. In that study, of the patients with a thyroid disease, 20 patients, representing 71.4%, took thyroid hormone preparations [21].

The aim of this cross-sectional study was to evaluate the frequency of dental allergens and potential co-factors especially hypothyroidism for patients with intraoral contact allergy.

Our hypotheses were:

1. There are no differences in the occurrence of metal or resin allergens in patients with symptoms of an intraoral contact allergy and those without.
2. Patients with a nickel allergy more often also have a cobalt allergy.
3. Patients with hypothyroidism have no higher risk of having a sensitization and/or allergy.

## 2. Material and Methods

The study was endorsed by the Ethics Committee of Leipzig University and is listed under the reference 075-15-09032015. The principles outlined in the Declaration of Helsinki in its latest version from the 64th WMA general meeting in October 2013 in Fortaleza (Brazil) were followed (World Medical Association 2013).

The study recruited participants from the university dental hospital allergy patients from April 2015 to April 2016. A healthy control group (CG) was formed comprising patients who visited their dentist for their annual dental check-up. Normally, we receive around 60–70 allergy patients in one year and decided to recruit twice as many controls, as this seemed practicable. Subjects in the study group (SG) were patients who had sensitization and/or allergies to dental materials with intraoral symptoms of a contact allergy. Exclusion criteria were: caries lesions, acute periodontics disorders or other pathological oral diseases. Additionally, patients who were dissatisfied with their dentures or who could not cope as well as those who developed symptoms, due to toxic, mechanical or microbiological stimuli, were excluded.

**Figure 1:**
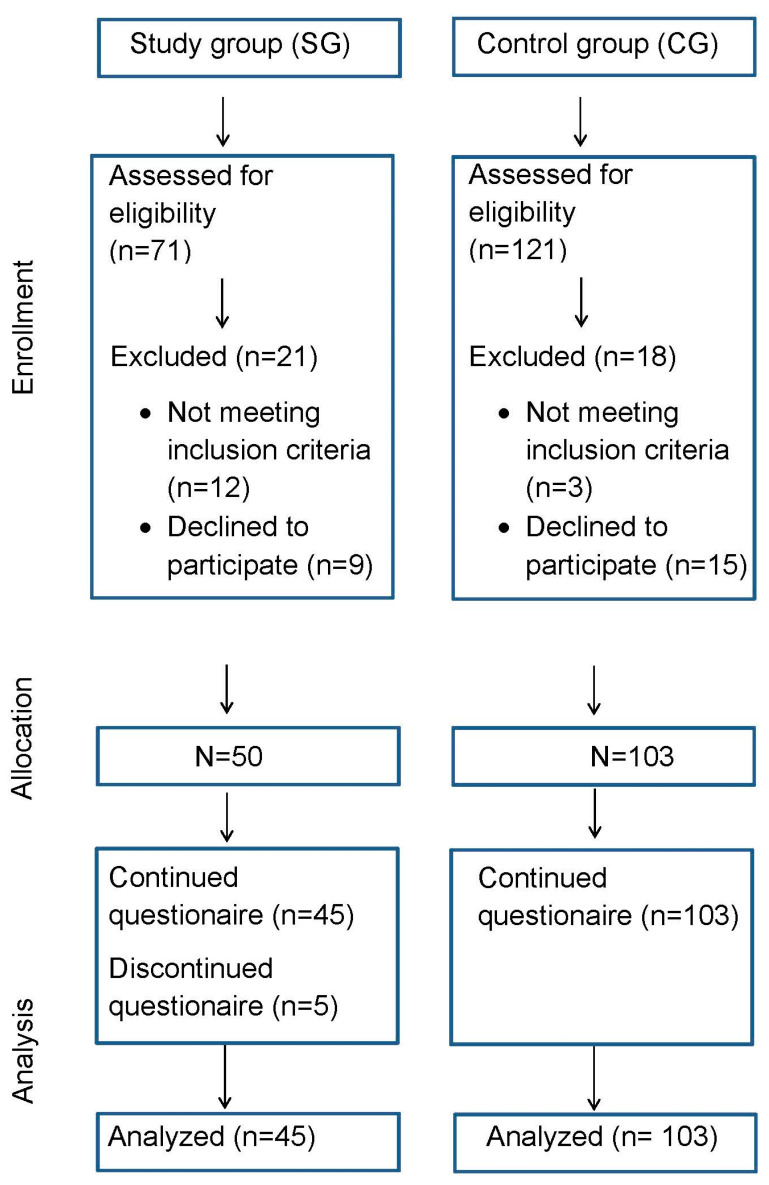
Modified Consort flow diagram.

The subjects of the CG had no symptoms of an intraoral contact allergy to dental materials, neither diseases of the oral mucosa nor acute complaints, caries lesions or acute periodontics disorders. All participants were at least 18 years of age and of both sexes. Participation was voluntary and the participants were informed about the study and its aims. Once they gave their consent, the subjects were sent the questionnaires via post and could send them back to study management using an addressed pre-stamped envelope (see Figure 1).

For the data collection, a newly designed “Allergy questionnaire” was developed. The questionnaire mostly dealt with the collection of allergen-specific data (allergy test, allergens) and information on the participant′s restorations [17,21]. Data on dentures and their materials used were collected based on the dental status, the material pass or the declaration of conformity of the laboratory-produced dentures. In addition to the collection of personal data, the general medical history was collected [21]. The anonymized data were divided into the following categories: dental status, allergen-specific data and medical history.

The statistical evaluation was carried out with SPSS 23.0 (SPSS Inc., Chicago, IL, USA). The frequency distributions were descriptive. Differences between the SG and CG were tested with the t-test or Mann–Whitney U-test. Associations were determined using regression analyses and Fisher′s Exact test. A type I (alpha) error of *p* < 0.05 was considered statistically significant and *p* < 0.001 was considered highly significant. As this study was of an exploratory nature, in order to generate hypotheses for possible future studies, no correction for multiple-testing was carried-out.

## 3. Results

The study included 50 subjects in the SG, of which 45 had complete data which could be used for the analysis (Figure 1). Data from the 103 participants in the CG were complete and all were used for this analysis (Figure 1). Table 1 shows the sociodemographic and dental restorations data. There were 38 (84.4%) females in the SG and 59 (57.3%) in the CG, and the median age was 65 (IQR: 55–71) in the SG and 50 (IQR: 39–59) in the CG.

The most commonly used direct restoration material was composite in both groups. The material amalgam was used in approximately 37% of cases in the CG. High-gold alloys were often used as a material for indirect restorations. In the area of fixed restorations, it was mainly ceramic veneer crowns with a base made of a non-noble metal alloy that had been used. Noble metal alloys were used much more frequently in the SG. In the SG, a fixed partial denture made of a noble-metal alloy and one of polyetheretherketone (PEEK) were used.

Table 2 shows the overview of the frequently found allergens in both groups. From the evaluation of the allergen-specific data, 33 of the patients in the SG show several different allergens. Dentally-relevant allergens, especially metals dominated in the SG. Looking at the metal allergies of all subjects of the SG and CG together, a total of 14.2% (21/148) of the subjects had a known nickel allergy. At the same time 6% (9) had a cobalt allergy and 2% (3) a palladium allergy. The Fisher′s Exact test showed that a nickel allergy was associated with a cobalt allergy (*p* < 0.001). A Palladium allergy also tended to occur more often together with a nickel allergy.

Data on the medical history show that although the average age in the SG was ten years higher, cardiovascular diseases were similarly distributed in both groups. Statistical differences were observed for fungal infections and thyroid diseases (Table 3). The analysis of the data shows a relationship between hypothyroidism and having an allergy. The evaluation of the data of the allergy consultation had previously suggested a connection between hypothyroidism and an allergy [21]. In the present study, 16 subjects (35.6%) in the SG were diagnosed with hypothyroidism. One subject did not provide any information and 28 subjects had no known thyroid dysfunction. In the SG, all subjects with a positive medical history of hypothyroidism were female. In the CG, 19 subjects (18.4%) had hypothyroidism, of which 17 (16.5%) were female and two (2%) were male. Thus, hypothyroidism occurred significantly more frequently (*p* = 0.049) in the SG than in the CG.

If the datasets of SG and CG are summed up, the data of 148 subjects can be analyzed. Of those, 82 (55.4%) reported at least one allergy (SG 37/82.2%, CG 45/43.7%). Overall, 68 (83%) with allergies were female and 14 (17%) were male. This difference was significant (*p* = 0.033). In total, 35 subjects of SG and CG had hypothyroidism. An association between thyroid dysfunction (hypothyroidism) and the occurrence of allergy was seen. A logistic binary regression analysis, adjusting for sex and age, showed that patients with hypothyroidism had a three times higher risk of having an allergy than patients without hypothyroidism (odds ratio = 2.994, 95% confidence interval 1.288–6.961, *p* = 0.011).

## 4. Discussion

The lack of information on the association between an intraoral contact allergy and hypothyroidism motivated us to carry out this cross-sectional observational study. The main aim was to find out whether there are co-factors for an intraoral contact allergy that are different between the two groups (study and control). The ratio of patients in the two groups was approximately 2:1.

In the present study, females had more adverse reactions to dental materials, which is also shown in the study by Vamnes et al. where 70% (208/296) of those affected were females [22]. Richter and Geier showed that in the stomatitis patients and in those who were tested for the clarification of a denture incompatibility, females and older patients predominated [23]. Patients with allergic complaints or material intolerances often have a long history of dental problems. Any existing restorations and the materials used for them are helpful for a clinical diagnosis. For instance, in our study, we saw that older, predominantly female patients with long-term dental history, had developed oral intolerance. In the SG, the median age was 65 years, and crowns, fixed partial dentures, removable partial dentures and complete dentures were more common, while in the CG (median age 50 years), direct restorations dominated. In the most recent German Oral Health Study (DMS V), 77.3% (747/966) had no removable partial nor complete dentures, and in the group of younger adults (35–45 years) only 2.7% were supplied with a removable denture [24,25]. Up to around 30 years ago, more high-gold alloys were used for fixed and removable partial dentures with a metal framework; however today, due to costs, non-noble alloys composed of cobalt-chromium are used. This can be seen from the information on dentures of the CG. Thus, younger adults (35–45 years) are coming into contact with cobalt-chromium alloys, e.g., for crowns and fixed partial dentures more often. This is also reflected in the increased contact sensitivities to cobalt compared to past data surveys [21]. Our hypothesis that there are no differences in the occurrence of metal or resin allergens in patients with symptoms of an intraoral contact allergy and those without, can therefore be rejected.

### 4.1. Occurrence of Cross-Allergies

Allergies to several substances can occur at the same time. A cross-allergy of nickel and palladium has been known to exist for a long time [26]. In the periodic table, both metals are in the same subgroup (VIII). Furthermore, reactions to palladium are observed in about 50% of patients with an allergy to nickel [27,28,29,30]. In the study by Rachmawati et al., 80% of patients (25) with a positive test reaction to nickel also showed sensitization to palladium salts [31]. In the study by Raap et al., (2009), all patients (100%, 10) with nickel sensitization also showed a positive patch-test response to palladium [32]. In the study by Muris et al., (2015), a cross-allergy between nickel and palladium was found only in patients with amalgam fillings and crowns. In particular, patients (10.2%, 91/906) with oral symptoms and other complaints had significant sensitizations to palladium and nickel. Dry mouth (xerostomia) and metallic taste were strongly associated with palladium and nickel sensitization in this study [33].

One third of all nickel allergy sufferers also show reactions to cobalt chloride, which is called a coupling allergy [34]. In the present study, allergic reactions to cobalt also occurred in SG′s nickel allergy sufferers. Cobalt is an element of the same subgroup (VIII) in the periodic table as nickel. In nature, cobalt and nickel coexist [35]. Cobalt is a potent allergen and is often described as a contact allergen [36]. As a result, sensitization to nickel and cobalt can occur simultaneously [37]. Whether this is a genuine, cross-allergic reaction has not yet been clearly clarified [37]. The study by Lidén et al. (2016) showed that 14% (92/656) of patients with contact dermatitis had a contact allergy to cobalt. Of these, 50% (46/92) had a solitary cobalt allergy [38]. Similar results have been reported in other studies [39,40,41].

Rarely have reactions to nickel, copper and chromium been observed [42]. In a separate meta-analysis, metallic allergens such as nickel, palladium and cobalt were common [43].

Isolated reactions to cobalt without simultaneous reactions to nickel and chromium are rather rare [34].

In the present study, the most frequent allergens in SG were metals. Allergies to metals and composites were higher in the SG compared to the CG. For instance, 14.2% (21) of all study participants (n = 148) had a nickel allergy, and in 18% of the SG a cobalt allergy based on all metal allergens could be detected. Our second hypothesis that patients with a nickel allergy have more often a cobalt allergy also, is supported by this study.

### 4.2. Allergies and Systemic Diseases

The hypothesis that patients with hypothyroidism had no higher risk of having an allergy is rejected. The occurrence of thyroid disease associated with allergic rhinitis, asthma and chronic urticaria has been described previously [44,45,46,47]. Recent studies also assume immunological interactions. Chronic idiopathic urticaria and autoimmune thyroid disease are characterized by the presence of anti-IgE and/or anti-IgE receptor antibodies and thyroid antibodies in some patients [48]. An increase in hypothyroidism has also been described in the clinical picture of oral lichen planus and oral lichenoid lesions. In the study by Rachmawati et al. (2015), there was an association between autoimmune diseases and sensitization to the dental metals nickel, palladium, gold, and mercury. No thyroid antibodies were found on sensitization to palladium, gold and mercury. In contrast, thyroid antibodies were detected in patients with nickel sensitization [31]. In the study by Muris et al. (2014), patients with palladium-containing dental alloys showed an increased epicutaneous test response and lymphocyte proliferation [49]. We saw a direct association between the occurrence of allergies and hypothyroidism in the present study (*p* = 0.047).

Studies with a higher number of participants need to be conducted to confirm our findings. Whether this is a true cross-allergy between nickel and cobalt should be clarified and to make a conclusive statement about which allergens might set off hypothyroidism and vice versa.

## 5. Conclusions

In the present study, not only was the common occurrence of nickel and palladium allergies together observed, but also that of nickel and cobalt allergies. We also saw an association (adjusted for sex and age) between hypothyroidism and an allergy. Patients with hypothyroidism were three times more likely to be allergic. Theoretically, sensitizations and allergies can occur to any ingredient in dental materials. It is therefore important for the dentist to know potential allergens in dental materials.

## Figures and Tables

**Table 1:** Sociodemographic and dental restorations data.

Personal Data	Study Group (n = 45)	Control Group (n = 103)
**Sex** Female	38 (84.4%)	59 (57%)
**Age in years—Median (IQR)**	65 (55–71)	50 (39–59)
**Profession**		
Pensioners	23 (51%)	18 (17%)
Non-academics	14 (31%)	48 (47%)
Academics	3 (7%)	11 (11%)
no profession	0	4 (4%)
no information	5 (11%)	22 (21%)
**Dental Restorations (not Mutually Exclusive)**		
direct restoration	31 (69%)	97 (94%)
indirect restoration	7 (16%)	11 (11%)
crowns	27 (60%)	52 (50%)
fixed partial dentures	20 (44%)	27 (26%)
dental implants	6 (13%)	4 (4%)
removable dentures (partial or complete)	25 (56%)	22 (21%)

**Table 2:** Allergens.

Allergen-Specific Data	Study Group (n = 45)	Control Group (n = 103)
Allergy pass	33 (73%)	23 (22%)
Allergy test	40 (89%)	49 (48%)
Patch test	32 (71%)	18 (17%)
Prick test	15 (33%)	44 (43%)
**Allergens Dental Restorations Materials**		
Nickel	11 (24%)	10(10%)
Mercury	10 (22%)	0
Cobalt	8 (18%)	1 (1%)
Palladium	3 (7%)	0
Copper	3 (7%)	0
Methacrylate	12 (27%)	1 (1%)
Composite	9 (20%)	0
Ceramic	2 (4%)	0
**Allergens Drugs**		
Antibiotics	13 (29%)	9 (9%)
Local anaesthetic	5 (11%)	1 (1%)
**Allergens other**		
Pollen (plants/grasses)	14 (31%)	28 (27%)
Food	11 (24%)	10 (10%)
Latex	8 (18%)	2 (2%)
House dust	8 (18%)	14 (14%)
Animal hair	4 (9%)	14 (14%)

**Table 3:** Results of medical history (SG…Study Group, CG…Control Group).

Diseases	SG n = 45 CG n = 103	Total	Female	Male	No Information	*p*-Value
Cardiovascular diseases	SG	20 44.4%	16 35.6%	4 8.9%	- -	0.454
	CG	39 37.9%	25 24.3%	14 13.6%	- -	
Thyroid disease (Hypothyroidism)	SG	16 35.6%	16 35.6%	- -	1 2.2%	**0.047**
	CG	19 18.4%	17 16.5%	2 2%	- -	
Fungal infections	SG	9 20%	9 20%	- -	1 2.2%	**<0.001**
	CG	2 2%	1 1%	1 1%	- -	
Pneumopathy	SG	7 15.6%	7 15.6%	- -	- -	0.118
	CG	6 5.8%	5 4.9%	1 1%	- -	
Autoimmune diseases	SG	7 15.6%	7 15.6%	- -	2 4.4%	0.218
	CG	5 4.9%	5 4.9%	- -	-	
Diseases of the internal organs	SG	7 15.6%	7 15.6%	- -	- -	**0.023**
	CG	5 4.9%	2 2%	3 2.9%	- -	
Neurological diseases	SG	5 11.1%	3 6.7%	2 4.4%	- -	**0.004**
	CG	1 1%	- -	1 1%	- -	
Blood disorders	SG	4 8.9%	4 8.9%	- -	- -	**0.015**
	CG	1 1%	-	1 1%	-	
Diabetes mellitus	SG	3 6.7%	2 4.4%	1 2.2%	- -	0.444
	CG	11 10.7%	6 6.8%	5 4.9%	-	
Insulin-dependent diabetes mellitus	SG	- -	- -	- -	- -	**<0.001**
	CG	6 5.8%	3 2.9%	3 2.9%	-	
Infectious diseases	SG	- -	- -	- -	- -	0.509
	CG	1 1%	1 1%	-	-	
